# Emicizumab in children: bleeding episodes and outcome before and after transition to Emicizumab

**DOI:** 10.1186/s12887-022-03546-1

**Published:** 2022-08-15

**Authors:** Hannah Glonnegger, Felicia Andresen, Friedrich Kapp, Stefano Malvestiti, Martin Büchsel, Barbara Zieger

**Affiliations:** 1 Department of Pediatrics and Adolescent Medicine, Division of Pediatric Hematology and Oncology, Medical Center, Faculty of Medicine, University of Freiburg, Freiburg, Germany; 2 Institute of Clinical Chemistry and Laboratory Medicine, Medical Center, Faculty of Medicine, University of Freiburg, Freiburg, Germany

**Keywords:** Emicizumab, Hemlibra, Children, Annual bleeding rate

## Abstract

**Purpose:**

Real-world data and study data regarding therapy with Emicizumab in pediatric cohorts with haemophilia A is scarce. Especially, data on previously untreated pediatric patients (PUPs) and minimally treated patients (MTPs) are missing.

**Methods:**

Thirteen pediatric patients with haemophilia A and treatment with Emicizumab were retrospectively evaluated for Annual Bleeding Rates (ABR) pre-and post-Emicizumab treatment. Safety data and data on management of minor surgery as well as laboratory results were collected. Additionally, we describe the clinical features of two PUPs and one MTP that are included in our cohort.

**Results:**

Median age at initiation of Emicizumab was 5.3 (range: 0.26–17.5) years, three patients were younger than one year at initiation of treatment with Emicizumab. Median follow-up time on Emicizumab was 23.8 (range: 0.7–40) months. Total ABR (*p* = 0.009) as well as spontaneous (*p* = 0.018), traumatic (*p *= 0.018), and joint (*p* = 0.027) ABR reduced significantly post-Emicizumab transition. Safety profile was favourable as only one local site reaction occurred; no cessation of treatment was necessary. Surgery was successfully performed in three patients receiving rFVlla pre- and post-surgery. Emicizumab trough levels showed a median of 43.2 μg/ml (range: 23.9–56.8) after three doses of 3 mg/kg and 51.9 μg/ml (range: 30.4–75) at first follow-up with 1.5 mg/kg.

**Conclusion:**

Emicizumab is safe and efficient in pediatric patients with and without inhibitors. More data on larger multicenter cohorts and especially on PUPs/MTPs are still needed.

## What is known-What is new?

### What is known

Emicizumab significantly reduces Annual Bleeding Rate (ABR) in adults and children and has a favourable safety profile in study setting. Data on young children, especially primary untreated patients (PUPs) and minimally treated patients (MTPs) is scarce.

### What is new

We add real-world data of young children, including primary untreated patients and a minimally treated patient and confirm reduction of ABR as well as a favourable safety profile in this younger cohort.

## Introduction

Haemophilia A is a severe bleeding disorder caused by deficiency or complete lack of coagulation factor Vlll (FVlll). Haemophilia A causes a chronic severe health burden due to its X-linked mode of inheritance which subjects male patients to severe bleeding episodes not only causing acute life-threatening conditions but also haemophilic arthropathy [1]. Lifelong prophylactic intravenous substitution of FVlll used to be the treatment of choice since the 1980s. In addition, patients may develop FVIII antibodies which require a frequent substitution of FVIII and/or of bypassing factors via a central venous line in the course of immune tolerance induction (ITI) [2].

In 2017, Emicizumab (Hemlibra®, Roche), a humanized bi-specific antibody was approved by the Food and Drug Administration (USA) for prophylactic treatment in patients with inhibitors. After demonstration of safety in non inhibitor patients (HAVEN 3 study [3]), approval for all haemophilia A patients followed. Emicizumab mimics the co-factor function of FVlll, bridging activated FlX and FX [4]. A great advantage of Emicizumab is its subcutaneous application (once weekly to once every four weeks), and therefore, the benefit especially in pediatric patients remains undisputed. Annual bleeding rates (ABR) of patients, formerly treated with prophylactic FVlll, reduced when treated with Emicizumab and the safety profile revealed no serious side effects under exclusive treatment with Emicizumab [4]. Several phase lll studies (HAVEN 1–4) further investigated different age cohorts, while only HAVEN 2 investigated pediatric patients (*n *= 85 aged < 12 years). All studies confirmed safety and efficacy of Emicizumab [3, 5–7]. Only few other studies have since investigated pediatric patients under prophylaxis with Emicizumab [8–12], especially data on PUPs and MTPs [13, 14] are limited.

For all non-factor replacement therapies like Emicizumab there is a potential risk of thrombosis. However, an increased risk of thrombosis in patients under sole Emicizumab prophylaxis or in combination with rFVII has not yet been described in clinical trials. Severe adverse events (death due to thrombotic microangiopathy) occurred under combination therapy with Factor Eight Inhibitor Bypassing Activity (FEIBA). Therefore, activated prothrombin complex concentrate is prohibited under Emicizumab therapy [5]. Other risks are anti-drug antibodies that were described in two pediatric patients (one with loss of efficacy of Emicizumab) [6] or breakthrough bleeding, mainly associated with trauma [8].

There is no standard procedure for surgery for patients with haemophilia and Emicizumab treatment in place. It is recommended to add FVlll according to the extent of the surgery in non-inhibitor patients and recombinant Factor seven a (rFVlla) in patients with inhibitors [15].

Conventional monitoring of therapy in haemophilia patients consists of FVlll activity analysis using one stage clotting assays. Emicizumab interferes with this laboratory testing and therefore, chromogenic FVlll activity analysis is necessary. The same applies for FVIII inhibitor testing. Monitoring of Emicizumab therapy is not generally recommended, but mean steady state concentrations for different dosing intervals in clinical trials were if administered once weekly: 51,1 μg/mL [3, 5], every two weeks: 46,7 μg/mL [3] and every four weeks: 38,3 μg/mL [7]. An exposure–response model showed that ABR decrease rapidly with an increasing Emicizumab plasma concentration. Concentrations above 30 μg/mL were predicted to provide clinically meaningful control of bleeding [16]. Activated partial thromboplastin time (aPTT) values generally normalize under treatment with low doses of Emicizumab. However, this does not reflect normalization of hemostasis [17].

As real-world data regarding pediatric patients and especially PUPs and MTPs on Emicizumab is still scarce, we investigated efficacy and safety of a cohort of 13 pediatric patients treated with Emicizumab in our outpatient clinic, focusing on the intrapersonal ABR before and after initiation of treatment. We also describe the clinical characteristics of two PUPs and one MTP of this cohort.

## Methods

### Patients

Retrospective data analyses of 13 pediatric patients with haemophilia A (12 severe, 1 moderate) and treatment with Emicizumab was performed at our section of pediatric hemostaseology. Two out of thirteen patients were PUPs, the youngest being 3 months old at the time of treatment initiation with Emicizumab and one patient was a minimally treated patient with less than 5 exposure days to FVlll. One patient was already > 18 years at the time of analysis, but < 18 years at the time of transition to Emicizumab.

Study inclusion criteria were: (1) Diagnosis of haemophilia A, objectively confirmed by FVlll activity of < 1% for severe and 1–5% for moderate haemophilia A and molecular genetic analysis, (2) treatment with Emicizumab, (3) regular follow-up examination and laboratory parameters at our department of pediatric hemostaseology, (4) informed consent to use data for evaluation and publication (approval of ethics committee of the University of Freiburg: 222/20 (registry study)).

Indication to transition to Emicizumab.

- Presence of inhibitors (high and low responder)
- Difficulties in venous access
- Preference of parents/patient due to easier form of application and/or hope for better compliance

Emicizumab was applied once weekly according to the manufacturer´s recommendation. The first dose of Emicizumab (3 mg/kg) was applied in our hospital to observe safety and side effects. The next two weekly loading doses of 3 mg/kg were administered by the children’s pediatrician. Emicizumab levels were screened before the fourth loading dose at our outpatient clinic. The following weekly dose consisted of 1,5 mg/kg Emicizumab.

FVlll substitution remained the treatment of choice for patients without inhibitors in case of breakthrough bleeds while on Emicizumab. For patients with inhibitors rFVlla was employed in case of trauma or surgery. No other bypassing agent was used for any patient on Emicizumab.

#### Data collection

The retrospective longitudinal patient chart review of clinically derived data included: patient demographics, previous FVlll therapy (prophylactic, ITI, PUP/MTP), treated bleeds in ABR before/after transition to Emicizumab subdivided into a) total ABR, b) spontaneous ABR, c) traumatic ABR, d) joint ABR, treatment duration before/after Emicizumab, reasons for transition to Emicizumab, comorbidities, motor activity level, side effects and treatment during surgery.

Of note, ABRs were calculated to account for different lengths of therapy with prophylactic FVlll and Emicizumab and only treated bleeds were considered for analysis. Additionally, different age groups (< 6 years vs. > 6 years) were compared regarding ABR.

Emicizumab levels were monitored after 3 doses of the loading dose of 3 mg/kg and at every follow-up appointment under therapy with 1,5 mg/kg. A modified, aPTT-based Factor VIII assay, calibrated against Emicizumab (r2 diagnostics) was used.

In addition, aPTT before and after transition to Emicizumab was analysed and FVIII inhibitor (Bethesda assay) was measured regularly.

FVlll activity using a chromogenic substrate assay based on bovine factors (which is unaffected by the presence of Emicizumab) was also monitored. Transaminases, electrolytes and creatinine were regularly assessed.

#### Statistical methods

Median values and ranges were reported, and nonparametric statistics were used to test for differences in continuous variables in terms of Inhibitor-status (Mann–Whitney-U-Test). Spearman's rank correlation was performed to measure the degree of association between the two continuously measured variables (age and ABR variables). The paired samples Wilcoxon rank test was used to compare paired data (ABR before – after treatment, < 6 years—> 6 years).

All *p*-values were 2-sided and values less than 0.05 were considered statistically significant. *P* values greater than 0.1 were reported as non-significant, whereas those between 0.05 and 0.1 were reported in detail. SPSS for Windows 28.0.1 (IBM Corp) was used for the statistical analysis of data.

## Results

### Patient demographics

Thirteen patients were included in the study. Twelve patients had severe haemophilia A, whereas one patient had FVlll-activities between < 1–2%. All patients received molecular genetic testing, confirming pathogenic mutations in the FVlll-gene in all patients. Their demographics and pre-Emicizumab treatment as well as description of reasons for initiating treatment with Emicizumab are presented in Table 1.

**Table 1:** Patient characteristics

**Patient number**	**Age (years)**	**Ethnicity**	**Inhibitors**	**Prior regimen**	**treatment duration Pre-Emicizumab**	**treatment duration Post-Emicizumab**	**Reason to transition to Emicizumab**	**Comorbidities**	**Motor activity level**
1	0.4	Caucasian	none	MTP^a^	0	0.7 mo	difficult venous access, parental decision	none	normal
2	2	Caucasian	none	PUP^b^	0	12 mo	difficult venous access, parental decision	none	normal
3	2	Caucasian	none	PUP^b^	0	0.9 mo	parental decision	none	normal
4	3	Caucasian	none	proph. FVlll^c^	4 mo	25 mo	difficult venous access, parental decision	none	normal
5	6	Caucasian	none	proph. FVlll^c^	4 y, 6 mo	12 mo	increasing problems with FVlll administration	autism	normal
6	9	Caucasian	none	proph. FVlll^c^	8 y, 1 mo	3 mo	parental decision, better feasibility (reduction of intravenous punctures and physician visits)	linguistic development delay	normal, very careful parents
7	12	African	none	proph. FVlll^c^	10 y, 4 mo	24 mo	parental decision	Juvenile idiopathic arthritis, asthma	normal
8	15	Caucasian	none	proph. FVlll^c^	12 y, 7 mo	3 mo	parental decision, reduced compliance due to puberty	hyperreactive bronchitis	normal
9	20	Caucasian	none	proph. FVlll^c^	16 y, 5 mo	28 mo	change of FVlll required due to availability	none	reduced
10	3	Caucasian	high-titre	proph. FVlll^c^	2 mo	29 mo	inhibitors	none	normal
11	6	Caucasian	high-titre	ITI^d^, FEIBA^e^	3 y, 2 mo	24 mo	inhibitors	ADHD^f^	high
12	9	Caucasian	high-titre	ITI^d^, FEIBA^e^	5 y, 5 mo	33 mo	inhibitors	none	normal
13	15	Caucasian	high-titre	failed ITI^d^, FEIBA^e^	11 y	40 mo	inhibitors	none	normal

^a^*MTP* Minimally treated patient, ^b^*PUP* Previously untreated patient, proph. ^c^*FVlll* Prophylactic treatment with FVlll, ^d^*ITI* Immune tolerance induction, ^e^*FEIBA Factor Eight Inhibitor Bypassing Activity* , ^f^*ADHD* Attention Deficit Hyperactivity Disorder

The median age at initiation of Emicizumab treatment was 5.3 (range: 0.26–17.5) years. Three patients were younger than one year at initiation of treatment with Emicizumab. Four out of thirteen (30.8%) patients were high-responding inhibitor patients, the other 9/13 (69%) patients did not have inhibitors.

Prior treatment consisted of prophylactic FVlll-substitution: for 7/13 (53.8%) patients (25–35 IU/kg thrice weekly), of whom in one patient (patient 10) Emicizumab plus ITI was started immediately after diagnosis of a FVIII-inhibitor. Two out of thirteen (15.4%) patients were on ITI when being switched to Emicizumab. ITI had failed in one patient (7,7%), and FEIBA had been used for prophylaxis before starting Emicizumab. Two (15.4%) patients were PUPs and 1 (7,7%) was a MTP. There were 2/13 (15.4%) patients with < 20 exposure days (patient 4, patient 10).

Median treatment time before transition to Emicizumab was 54 (range: 0–197) months and median follow-up time on Emicizumab was 23.8 (range: 0.7–40) months.

### Efficacy

Median total ABR pre-Emicizumab was 0.25 (range: 0–4) compared to 0 (range: 0–0.5) post-Emicizumab (*p* = 0.009), (Table 2).

**Table 2:** Annual Bleeding Rate and laboratory results before and after transition to Emicizumab

Patient number	ABR total Pre-Emicizumab	ABR total Post-Emicizumab	ABR spont.^a^ Pre-Emicizumab	ABR spont.^a^ Post-Emicizumab	ABR traumatic Pre-Emicizumab	ABR traumatic Post-Emicizumab	ABR joint Pre-Emicizumab	ABR joint Post-Emicizumab	Emicizumab (μg/ml) level after 3 doses (3 mg/kg)	Emicizumab level at first follow-up (1.5 mg/kg)
1	4	0	0	0	4	0	0	0	56.8	56.8
2	0	0	0	0	0	0	0	0	34.4	68.4
3	1	0	0	0	1	0	0.5	0	51.9	51.9
4	0	0	0	0	0	0	0	0	49.9	75
5	0.16	0	0	0	0	0	0	0	37.4	66.3
6	1.5	0	0.25	0	1.13	0	0.5	0	43.2	51.6
7	0.08	0	0.1	0	0	0	0	0	41.4	49.9
8	0.53	0	0.16	0	0.24	0	0.16	0	48.4	51.8
9	0.4	0.5	0.18	0	0.24	0.06	0.18	0	43.7	63.5
10	0	0	0	0	0	0	0	0	35.1	56.1
11	0.33	0	0.33	0	0.33	0	0.33	0	23.9	30.4
12	0.11	0	0.18	0	0	0	0	0	39.6	38.1
13	1.55	0	1	0	0.27	0	0.63	0	52.1	41.7
**Median (Min–Max)**	**0.25** **(0–4)**	**0** (0–0.5)*	**0.05** **(0–1)**	**0** (0–0)*	**0.23** **(0–4)**	**0** (0–0.06)*	**0.06** **(0–0.63)**	**0** (0–0)*	**43.2** **(23.9–56.8)**	**51.9** **(30.4–75)**

^**a**^*spont* Spontaneous

^*^p < 0.05

In addition, median spontaneous ABR (*p* = 0.018), traumatic ABR (*p* = 0.018), and median joint ABR (*p* = 0.027) were significantly reduced after transition to Emicizumab.

Twelve (92.3%) of the patients experienced no bleeds after transition to Emicizumab. There was a traumatic bleed in one patient on Emicizumab.

If patients aged > 6 years were compared to patients < 6 years, the only significant difference occurred in spontaneous ABR, which was significantly reduced post-Emicizumab in younger patients (*p* = 0.016).

There was no correlation between age and ABR. There was a tendency towards higher spontaneous ABR before Emicizumab in patients with inhibitors compared to non-inhibitor patients (*p* = 0.104), however it did not reach significance level.

One patient presented with a target joint before transition to Emicizumab (patient 13). After transition to Emicizumab, no novel bleed into that joint occurred. The latest clinical examination of that joint was without pathological findings.

### Surgery

Three inhibitor-patients received minor surgery, all regarding removal of their central venous lines, while one patient additionally underwent keloid scar removal. All of them received one pre-operative dose of rFVlla (~ 100 µg/kg). Two patients received two additional post-operative FVIIa substitutions after surgery and on the first postoperative day. The patient with keloid scar removal received a total of 6 postoperative doses rFVlla until 3 days post-surgery. No peri- or postsurgical bleeding occurred.

### PUPs/MTPs

The two PUPs and one MTP are presented in detail.

#### Patient 1 (PUP)

A 9-month-old boy was referred to our outpatient department because of recurrent hematoma. A diagnosis of severe haemophilia A was made based on a reduced FVlll activity (< 1%). No other bleeding event had occurred. The parents received comprehensive and repeated education and opted for Emicizumab as primary prophylaxis, since i.v. injections were very challenging in this patient. After head trauma, the patient once received plasmatic FVlll in addition to his Emicizumab prophylaxis and did not develop any bleeding. Molecular genetic testing revealed a hemizygous variant of the FVlll-gene (c.[5188A > G];[0] p.(Met1730Val)). Follow-up examinations (12 months) did not show any signs of inhibitors against FVlll and no bleeding had occurred.

#### Patient 2 (PUP)

A 3.5-year-old boy was diagnosed with haemophilia A after a traumatic joint bleed of the ankle. Medical history only revealed one extended mucocutaneous bleeding at the age of 1.5 years. FVlll activity was < 1% and 2%, respectively. Molecular genetic analysis revealed a hemizygous FVlll-gene variant (c.[5339C > T];[0] p.(Pro1780Leu)). Because of the increased bleeding symptoms, a prophylaxis using Emicizumab was initiated based on parental preference.

Follow-up examinations (2 months) did not show any FVIII-inhibitors and no bleeding occurred. No FVlll was administered until inclusion to the study.

#### Patient 3 (MTP)

At the time of diagnosis, a 3-month-old boy had already suffered from postnatal cephalic hematoma that required transfusion of red blood cells, but no further diagnostics were initiated. After persistent bleeding following frenectomy, coagulation studies were initiated, and severe haemophilia A was diagnosed. The initial treatment of the acute bleeding consisted of 5 substitutions with plasmatic FVlll (30 IU/kg). Because venous access was very difficult, the parents asked for prophylactic treatment with Emicizumab, which was initiated shortly after. In addition, the boy received prophylactical FVlll-substitution once weekly (30 IU/kg). Molecular genetic testing revealed a missense mutation of the FVlll-gene (c.[1043G > A];[0] p.(Cys348Tyr)).

Follow-up examination after 3 months did not show any signs of inhibitors against FVlll and no other bleeding episodes occurred so far.

### Emicizumab levels.

Emicizumab trough levels after three doses of 3 mg/kg (= before 4^th^ dose) showed a median of 43.2 μg/ml (range: 23.9–56.8) and at first follow-up (with 1.5 mg/kg) increased to 51.9 μg/ml (range: 30.4–75). Median follow-up Emicizumab testing was performed after 67 days (range: 42–163), (Table 2).

### aPTT levels

aPTT decreased inversely to Emicizumab levels from median 110 s. (range: 71- > 140) to 27 s. (range: 23–40).

### FVlll activity levels

Regarding FVlll activity levels using the chromogenic assay with bovine factors, all patients had FVlll activity levels of < 3,3% after transition to Emicizumab.

### Safety

**Fig. 1:**
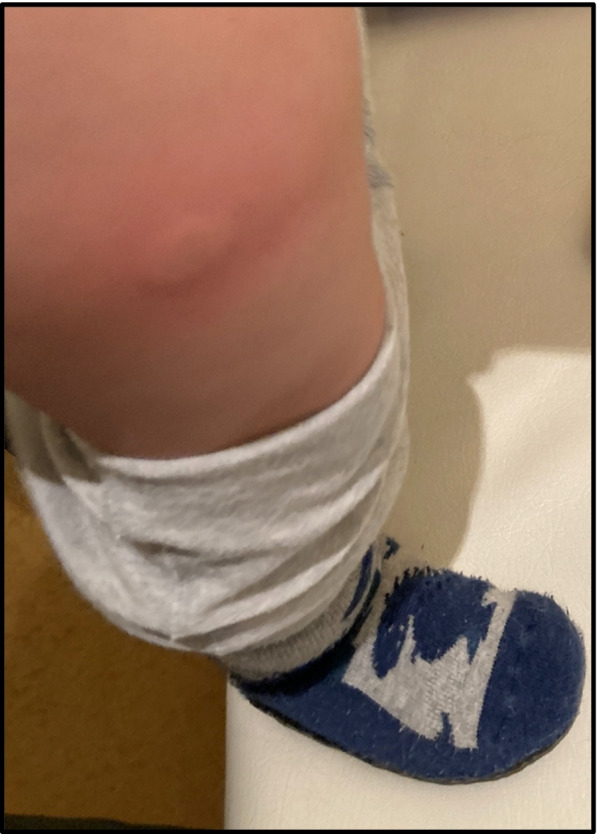
Local site reaction after subcutaneous injection of Emicizumab

Two patients presented with local skin reaction at the injection site after the first Emicizumab dose. One of the two patients recurrently showed urticaria at injection site after every injection (Fig. 1). We performed skin prick test to exclude allergic reaction to Emicizumab or other contents of the medication. No skin reaction occurred during prick test. Deeper subcutaneous injection improved the clinical symptoms remarkably. No patient developed thrombosis, thrombotic microangiopathy, renal failure, liver function impairment or blood count abnormalities. No cessation of treatment was necessary in any patient due to side effects.

## Discussion

Data on efficacy and safety of Emicizumab in children, especially in young children < 3 years and PUPs is still scarce. Between 2019–2021 several publications have reported the use of Emicizumab in children, including about 40 children under the age of 3 years [8, 9, 11–13]. All studies showed similar results regarding reduction of ABR, safety and laboratory results as adult cohorts. But especially PUPs demand special attention, because avoiding FVlll exposure might impose a higher risk of inhibitor development on these patients in case of breakthrough bleeding or surgery [18].

This current study provides additional evidence on efficacy and safety of Emicizumab in 5 children < the age of 3 years, two PUPs and one MTP. Our data show that the ABR significantly reduced under Emicizumab, regardless of age and inhibitor status. There were no spontaneous breakthrough bleeds during the long follow up-time of 24 months in our study. Other studies presented data of up to 50% breakthrough bleeds on Emicizumab [8].

Regarding PUPs, there is still no standardized recommendation regarding primary prophylaxis with Emicizumab in very young children. In 2020, the Society for Thrombosis und Hemostasis (GTH) published a guidance regarding the use of Emicizumab that stated a 92% agreement rate to the statement that “The decision to use Emicizumab in small children, especially PUPs, has to be made on an individual base” [15]. Published studies of pediatric patients on Emicizumab included three PUPs and one minimally treated patient in a total cohort of 52 individuals [14] and one PUP in the HOHOEMI study [13]. Both studies reported no other safety profiles or differences in efficacy compared to the other pediatric studies.

Therefore, our study adds to the data that, although haemostasis may still be developing in young children and FlX -activities may be low during the first months of life, efficacy in terms of reduction of ABR of Emicizumab is nevertheless comparable to adults. In addition, the same dosing can reach stable levels of Emicizumab. We were able to confirm this finding in children under the age of 3 years. We also report on two PUPs and one MTP that experienced no complications while being treated with Emicizumab. This was independent of whether Emicizumab was the initial treatment, initiated upon occurrence of inhibitors or administered in combination with FVlll.

Although this current study provides crucial information about treatment of PUPs, future studies, e.g. the Haemophilia Inhibitor Prevention Trial (NCT04303559) on PUPs, will provide more extensive data on this particular group and add valuable insight. Additionally, the HAVEN 7 study is ongoing, entitled: A Study to Evaluate the Efficacy, Safety, Pharmacokinetics, and Pharmacodynamics of Subcutaneous Emicizumab in Participants From Birth to 12 Months of Age With Hemophilia A Without Inhibitors [19].

Currently, there is still an ongoing debate on eradication of inhibitors in the era of Emicizumab. Most experts argue that inhibitor eradication by ITI should be initiated to be able to use FVlll products in case of breakthrough bleeds or surgery. Others argue that Emicizumab is efficient to prevent bleeding and therefore, ITI is not mandatory [20–22]. The German expert panel of the GTH recommended that one treatment trial with ITI should be offered to patients on Emicizumab [23]. Our cohort consisted of four high-titre inhibitor patients of whom three had already received several years of ITI long before transition to Emicizumab. One patient was transitioned to Emicizumab and additional ITI according to the Atlanta-Protocol (3 × 100/IU/kg weekly) immediately after diagnosis of high-titre inhibitors. Due to very difficult venous access and parental decision, ITI was terminated in this patient after a total of 2,5 years on ITI. Overall, none of our patients had a breakthrough bleed and according to patients and parents perspective, quality of life improved significantly.

In our cohort injection site reactions including minor skin reddening resembled previous reports [6]. We used a skin prick test to exclude acute allergic reaction to Emicizumab. Deeper deposition of the Emicizumab (still on the subcutaneous level) led to no more local site reaction in this patient. No other safety issues occurred during the study period.

We show that minor surgery in patients receiving Emicizumab prophylaxis is safe and that no bleeding or thrombosis occurred, even if few doses of rFVlla were administered additionally [5]. Nevertheless, treatment strategies in case of major trauma or other major surgery should still be investigated in further studies.

Lastly, we demonstrate that efficient Emicizumab levels were reached already after 3 doses of 3 mg/kg and were stable at follow-ups in most patients. This went along with the already well documented effect of shortening the aPTT [8, 17].

This study has some limitations, especially due to its retrospective design and limited number of patients. This makes statistical analysis vulnerable for outliers and generalization of results must be considered carefully. One example is patient 1, who experienced two bleeds in 6 months and therefore has an ABR of 4, which is very high compared to our cohort in total. The retrospective design also limited the analysis to treated bleeds alone, as only these bleeds are thoroughly documented in patient data. Additionally, the follow-up period in some patients is short and there was only one bleed on Emicizumab. Therefore, at the time of this study we are unable to contribute any information on FVlll inhibitor development in case of bleeding on Emicizumab.

In the future, there might be patients in this cohort being treated with different dosing regimen (e.g. once every four weeks) and further studies with other dosing regimens may offer new insights.

In conclusion, we report longitudinal data on a small pediatric cohort including two PUPs and three MTPs that were treated safely and efficiently with Emicizumab. The debate on ITI and treatment decision making on PUPs/MTPs in the era of Emicizumab must be validated by larger cohorts.

## Data Availability

All data generated or analyzed during this study are included in this published article.

## Abbreviations

ABR: Annual bleeding rate
aPTT: Activated partial thromboplastine time
FVlll: Factor eight
FEIBA: Factor Eight Inhibitor Bypassing Activity
GTH: Gesellschaft für Thrombose und Hämostase (Society for Thrombosis und Hemostasis)
ITI: Immune tolerance induction
MTP: Minimally treated patient
PUP: Primary untreated patient
rFVlla: Recombinant Factor FVll a
